# Effect of Quercetin-Doped Hydrogen Peroxide Gels on Enamel Properties: An In Vitro Study

**DOI:** 10.3390/gels11050325

**Published:** 2025-04-27

**Authors:** Renata de Oliveira Alves, Gabriel Pereira Nunes, Tamires Passadori Martins, Priscila Toninatto Alves de Toledo, Matheus Henrique Faccioli Ragghianti, Alberto Carlos Botazzo Delbem

**Affiliations:** Department of Preventive and Restorative Dentistry, Araçatuba School of Dentistry, São Paulo State University—UNESP, Araçatuba, 16015-050, SP, Brazil; ro.alves@unesp.br (R.d.O.A.); gabriel.p.nunes@unesp.br (G.P.N.); tamires.passadori@unesp.br (T.P.M.); priscilatoninatto@hotmail.com (P.T.A.d.T.); matheus.ragghianti@unesp.br (M.H.F.R.)

**Keywords:** tooth bleaching, hydrogen peroxide, quercetin, flavonoids, antioxidants, dental enamel

## Abstract

This in vitro study evaluated the effects of incorporating quercetin (QC) at varying concentrations (0.25%, 0.5%, and 1%) into a 35% hydrogen peroxide (H_2_O_2_) bleaching gel on esthetic outcomes, enamel hardness and roughness, and H_2_O_2_ transamelodentinal diffusion. Bovine enamel/dentin discs *(n* = 180; 12/per group for each analysis) were allocated into five groups: (1) negative control (NC), (2) 35% H_2_O_2_ (HP), (3) HP + 0.25% QC, (4) HP + 0.5% QC, and (5) HP + 1% QC. Treatments were applied for 40 min per session across three sessions with 7-day intervals. Color changes were evaluated using the CIELab* color system (ΔE_ab_), with further analysis performed using the CIEDE2000 formula (ΔE_00_) and the whitening index (ΔWI_D_). Enamel surface hardness, roughness, cross-sectional hardness, and H_2_O_2_ diffusion were also evaluated. Data were analyzed using ANOVA, followed by the Student–Newman–Keuls test, with statistical significance set at *p* < 0.05. All experimental gels resulted in significant color changes (*p* < 0.001), with similar ΔE_ab_, ΔE_00_, and ΔWI_D_ across QC groups. The HP group showed greater reductions in hardness and increased roughness compared to others (*p* < 0.0001), while the HP/1%QC group resulted in no statistically significant alterations under the tested conditions. H_2_O_2_ diffusion was significantly greater in the HP group, while it was notably lower in the HP/1%QC group (*p* < 0.05). The incorporation of 1% quercetin into a 35% H_2_O_2_ gel maintains its bleaching efficacy while protecting enamel properties and reducing hydrogen peroxide diffusion. Quercetin-enriched H_2_O_2_ gels may enhance bleaching safety by protecting dental tissues while maintaining esthetic benefits.

## 1. Introduction

Dental bleaching has gained widespread popularity among patients seeking to enhance tooth color esthetics [1,2], with the in-office technique being one of the most frequently employed methods in esthetic dentistry [3]. Despite the shorter application time of in-office bleaching gels compared to at-home treatments, several studies over recent decades have consistently confirmed the efficacy of in-office procedures [1,3,4,5,6]. Hydrogen peroxide (H_2_O_2_) remains the most widely used bleaching agent in these treatments [3,4], due to its ability to oxidize organic dental structures [1,3,7].

Although generally satisfactory esthetic results have been achieved, dental bleaching still faces challenges, such as in providing a comfortable, fast, and effective technique for patients [3,6] while also minimizing dental sensitivity during and after the procedure—one of the primary barriers to treatment adherence [1,3]. Additionally, studies have reported that bleaching can negatively affect enamel’s mechanical properties, leading to reduced hardness, changes in surface roughness, and the high transamelodentinal diffusion of H_2_O_2_ [8,9,10]. To address these concerns, various active ingredients have been tested in bleaching gel formulations to prevent or minimize adverse effects during dental bleaching [9,11,12,13].

The use of antioxidant substances in whitening treatment has been widely investigated due to their potential to neutralize or reverse the oxidative effects of H_2_O_2_ [14,15,16,17]. Among these substances, flavonoids, known for their antioxidant and anti-inflammatory properties, stand out, with quercetin being the main flavonoid in the human diet [16]. Its antioxidant action is due to its ability to sequester free radicals and chelate metal ions, neutralizing oxidative stress [18]. Moreover, quercetin offers several therapeutic benefits, such as the inhibition of carcinogenesis [19], cardioprotective effects [20], and nephroprotective effects [21]. In dentistry, quercetin has shown promise when incorporated into various dental materials, helping to improve their properties and bioactivity on dental substrates. Studies indicate that the addition of quercetin can enhance the performance of these materials, due to its antioxidant and anti-inflammatory properties, helping to protect and regenerate dental tissues [22,23,24,25,26].

Regarding the performance of quercetin in dental bleaching, its use after whitening therapy resulted in improved mechanical properties and greater enamel bond strength [22,27]. In addition, flavonoids, such as quercetin, have demonstrated the ability to reduce the cytotoxic effect of H_2_O_2_, reducing the damage caused by the oxidative stress associated with this agent [28]. However, to the best of our knowledge, studies on the efficacy of flavonoids incorporated directly into bleaching gels remain scarce. The addition of quercetin to bleaching gel could potentially inhibit or attenuate the changes in the dental substrate caused by the high oxidizing potential of H_2_O_2_ and the release of reactive oxygen species. Based on this, this study aimed to evaluate, in vitro, the addition of quercetin in different concentrations (0.25%, 0.5% and 1%) to 35% H_2_O_2_, analyzing the esthetic effect, microhardness, enamel roughness, and H_2_O_2_ transamelodentinal diffusion. The study’s null hypothesis was that the addition of quercetin would not influence the bleaching effect, microhardness, enamel roughness, or H_2_O_2_ diffusion in the bleaching gels tested.

## 2. Results and Discussion

**Figure 1:**
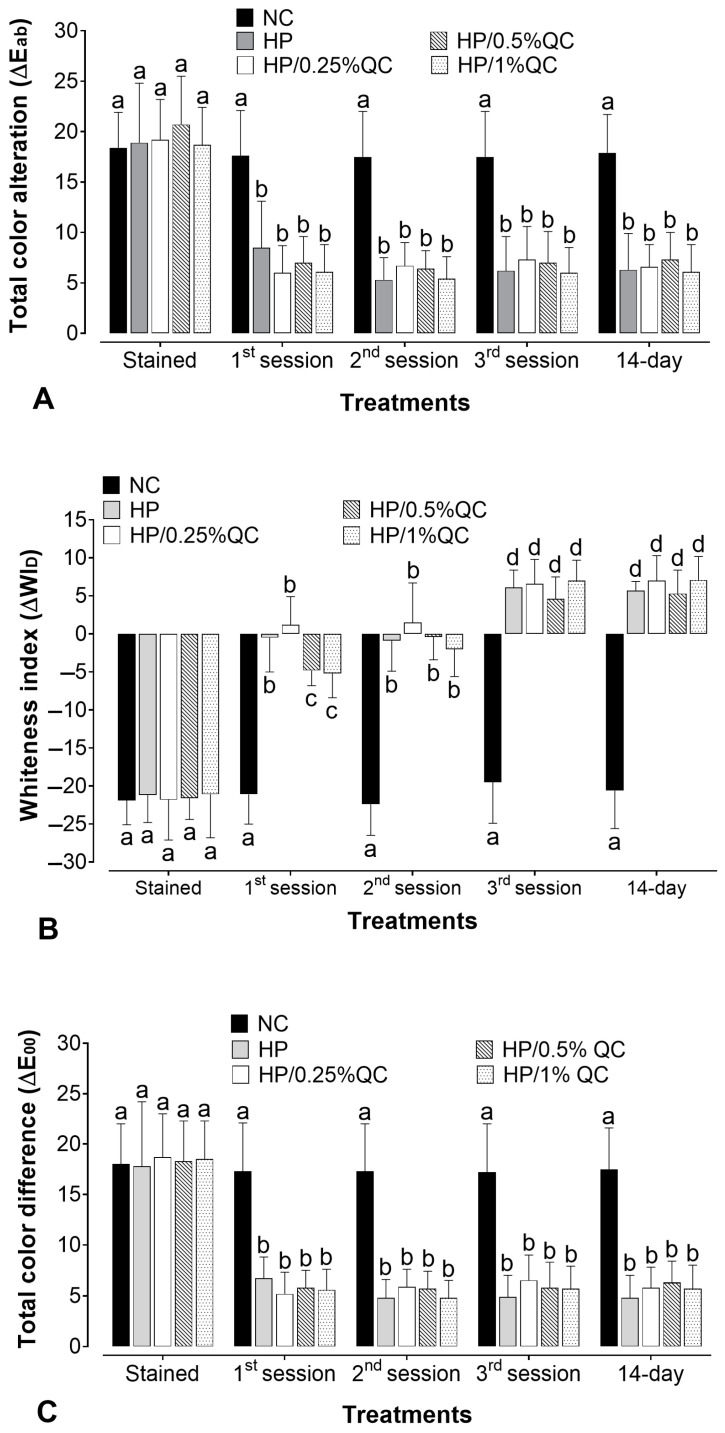
Mean values for the alteration of (**A**) total color alteration (ΔE_ab_), (**B**) the whitening index in dentistry (∆WI_D_), and (**C**) color alteration by CIEDE2000 (ΔE_00_) according to bleaching gels and the time of analysis (*n* = 12). Different subscript lowercase letters indicate statistical differences between bleaching gels at each time of analysis and between times of analysis for each experimental group (Student-Newman-Keuls; *p* < 0.05).

The bleaching gels promoted a significant color change (ΔE_ab_) after the treatment sessions (*p* < 0.05), except for the negative control group, which showed no significant color change at any of the times evaluated (*p* > 0.05). For H_2_O_2_-containing agents, the whitening effect was more evident after the first session, with a steady progression in subsequent sessions (Figure 1A). When comparing the different bleaching gels, there was no statistically significant difference in whitening efficacy (ΔE_ab_) between them (*p* > 0.05). Furthermore, with respect to the Whiteness Index for Dentistry (ΔWI_D_), the H_2_O_2_-based groups showed gradual and continuous chromatic improvements throughout the treatment sessions, resulting in visibly whiter samples. Additionally, no significant differences were observed among the groups, even with the varying concentrations of quercetin (*p* > 0.05) (Figure 1B). For ΔE_00_, all H_2_O_2_-based treatments promoted clinically perceptible color changes (ΔE_00_ > 0.8), with similar whitening effectiveness across all time points (*p* > 0.05) (Figure 1C). Across all parameters analyzed (ΔE_ab_, ΔWI_D_, and ΔE_00_), the bleaching agents sustained a stable effect up to 14 days after treatment completion (*p* > 0.05).

The bleaching treatment significantly reduced the surface hardness values in the experimental groups, except for the HP/1%QC group, which showed similar results to the negative control (Table 1). The addition of 0.25% and 0.5% quercetin to the bleaching gels resulted in similar hardness values (*p* > 0.05), but both reduced the loss of hardness when compared to the HP group (*p* < 0.001). Similarly, the surface roughness was significantly altered by treatments with HP, HP/0.25%QC, and HP/0.5%QC gels (*p* < 0.05). The negative control and HP/1%QC groups maintained roughness values similar to those observed before the bleaching procedure (*p* > 0.05; Table 1). For both variables (surface hardness and roughness), the HP gel caused the greatest changes in the enamel (SH, *p* < 0.001; %SH, *p* < 0.001; Ra, *p* < 0.001), while the HP/1%QC gel did not cause significant changes in the hardness and roughness of the enamel surface (*p* > 0.05; Table 1).

**Figure 2:**
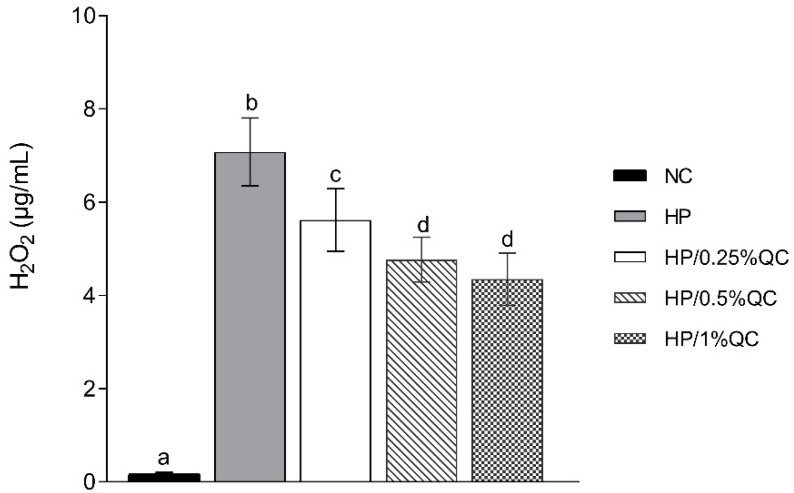
Mean values (SD) of transamelodentinal diffusion of H_2_O_2_ according to treatments (*n* = 12). Distinct superscript lowercase letters indicate statistical differences among bleaching gels in each variable (Student–Newman–Keuls test, *p* < 0.001).

**Figure 3:**
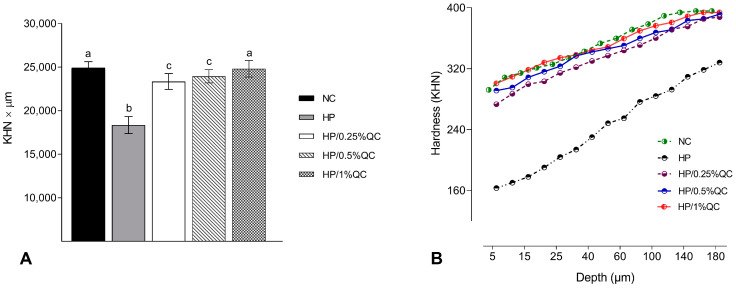
Cross-sectional hardness analysis. (**A**) Mean values (SD) of cross-sectional hardness (KHN × µm). Distinct superscript lowercase letters indicate statistical differences among experimental groups (Student–Newman–Keuls test, *p* < 0.001). (**B**) Cross-sectional hardness profiles at different depths in the enamel (*n* = 12) according to the experimental groups.

Regarding the transamelodentinal diffusion of H_2_O_2_, the HP bleaching gel showed the highest H_2_O_2_ diffusion, while the HP/1%QC and HP/0.5%QC groups showed the lowest values, with reductions of 38% and 33%, respectively, compared to the HP group (*p* < 0.001; Figure 2). For cross-sectional hardness, significant reductions were observed in the HP-, HP/0.25%QC-, and HP/0.5%QC-treated groups, with the most pronounced decrease in hardness occurring at depths of 5–50 μm within the dental enamel (Figure 3A,B). The HP gel showed lower ΔKHN across all evaluated depths. In contrast, the HP/1%QC bleaching gel exhibited ΔKHN values similar to those of the negative control group, as shown in Figure 3B.

New approaches to the development of whitening gels have focused on incorporating bioactive agents to protect dental tissues from the adverse effects of hydrogen peroxide [10,11,12,13]. Antioxidant substances are gaining prominence for their ability to neutralize free radicals generated during the whitening process [22,29]. These formulations aim to enhance biocompatibility, offering safer and more effective treatments with a reduced risk of damage to dental tissues. This in vitro study assessed the influence of adding distinct concentrations of quercetin (0.25%, 0.5%, and 1%) to bleaching gels containing 35% hydrogen peroxide, focusing on esthetic efficacy, mineral loss, and enamel roughness. The null hypothesis that quercetin incorporated into bleaching gels would not alter the whitening effect was accepted, as quercetin did not affect the gel’s whitening performance. However, the null hypotheses that quercetin would not influence hardness, enamel roughness, or transamelodentinal diffusion were rejected. The presence of quercetin was found to reduce (at concentrations of 0.25% and 0.5%) and prevent (at 1% concentration) demineralization, changes in enamel roughness, and H_2_O_2_ transamelodentinal diffusion.

In the present study, the most pronounced color changes were observed during the initial bleaching session, corroborating previous findings that hydrogen peroxide induced a greater oxidation–reduction effect upon first exposure, with smaller chromatic changes observed in subsequent sessions [30,31,32]. Generally, all H_2_O_2_-based bleaching gels demonstrated equivalent whitening efficacy, regardless of the chromatic evaluation methods used (ΔE_ab_; ΔWI_D_; and ΔE_00_). Furthermore, the color change was deemed detectable by the naked eye as the ΔE_ab_ values exceeded values surpassing 3.3 (Figure 1A) [33]. Additionally, ΔWI_D_ values (Figure 1B) were positive after completing the whitening protocol, and ΔE_00_ was also higher than 3 (Figure 1C). It is known that a ΔE_00_ > 0.8 is required to detect a clinically perceptible color change [34]. Thus, within the parameters evaluated, and regardless of the concentration of quercetin in the bleaching gel, all hydrogen peroxide-based experimental groups exhibited a similar whitening capacity.

As previously noted by Łopusiewicz et al. [35], there is some concern regarding the yellowish coloration of quercetin and its potential impact on chromatic parameters. Consequently, the potential for quercetin to alter or reduce the whitening effect of the gels, as well as the risk of dental staining, were considered. However, none of the three quercetin concentrations tested (0.25%, 0.5%, and 1%) compromised the hydrogen peroxide bleaching effect (Figure 1). It is crucial to emphasize that the duration of exposure to the gel during each bleaching session is relatively short (40 min/per session), suggesting that it may be insufficient to cause dental staining. Furthermore, the application of antioxidant solutions following dental bleaching has been described in the literature for its potential to maintain an intact adhesive interface, neutralize H_2_O_2_, and provide satisfactory enamel surface properties, without affecting the color stability of restorative materials [36,37]. Although no studies have specifically evaluated the incorporation of quercetin into bleaching gels, a recent study by Lin et al. [23] reported that quercetin applied as a pre-treatment solution did not interfere with the whitening effect on dentin, which is consistent with our findings. Additionally, we did not observe any color regression 14 days following the completion of the whitening treatment in the quercetin-containing groups. These results encourage future clinical investigations that may offer a potential whitening alternative to reduce the harmful effects of bleaching treatments while maintaining their clinical effectiveness.

Regarding the other enamel properties evaluated, the HP group showed the greatest decrease in SH and ∆KHN values, alongside the highest increase in surface roughness (Ra) compared to the other experimental gels (Table 1; Figure 3). These alterations are possibly related to agents containing hydrogen peroxide, which, when they come into contact with dental enamel, have the ability to induce modifications to, or the dissolution of, mineralized structures [10,38]. Conversely, the experimental groups containing quercetin showed smaller changes in hardness and roughness, with the 1% concentration demonstrating no significant alterations. This effect is possibly related to the ability of this flavonoid agent to reverse the damage caused by oxidative stress induced by the procedure [39]. Additionally, quercetin’s potential to prevent demineralization and surface roughness may be related to its ability to increase acid resistance [40], promote remineralization [40,41,42,43], and decrease erosive tooth wear [40,44,45]. These factors support the lower H_2_O_2_ transamelodentinal diffusion observed in quercetin-containing gels, particularly at concentrations of 0.5% and 1% (Figure 2). Thus, quercetin may contribute to the preservation of dental structures by modifying their permeability and limiting the penetration of substances through enamel and dentin.

Quercetin is a bioactive molecule widely recognized for its role in promoting various biological processes [18]. The findings of this study corroborate previous research demonstrating quercetin’s potential to enhance the mechanical properties of dental tissues [22,42,45]. Its application has proven effective in remineralizing enamel caries lesions and improving enamel surface topography [41]. In dentin, quercetin stabilizes the collagen matrix, acting as a barrier that prevents acid penetration, thereby inhibiting demineralization [40]. Furthermore, its antioxidant properties suggest a reduction in the diffusion of H_2_O_2_ into pulp tissue and in the penetration of H^+^ ions generated by H_2_O_2_ dissociation, offering enhanced protection to dental structures during bleaching. This supports the findings of the present study. Additionally, quercetin effectively suppresses free radicals by inhibiting the formation of superoxide ions, hydroxyl radicals in the Fenton reaction, and lipid peroxide radicals [46]. Its antioxidant capacity is further enhanced by its ability to react with these radicals and form iron complexes, which block the catalysis of reactive oxygen species [18]. Due to its non-toxic nature and its ability to inhibit free radical processes at various stages, quercetin is considered a highly potent natural antioxidant [18,46].

Moreover, previous investigations have demonstrated that the use of quercetin, both before [23] and after bleaching [22], significantly enhances the bond strength of restorative materials. These findings suggest that quercetin plays a crucial role in reversing oxidative processes in mineralized dental tissues, thereby highlighting its importance not only in preserving the mechanical properties of enamel but also in optimizing the performance of restorative procedures immediately following bleaching. Preserving enamel’s structural integrity after exposure to hydrogen peroxide is essential, as bleaching may impair the adhesion of restorative materials due to oxidative stress. Antioxidant solutions, such as quercetin, have shown great promise in reducing the adverse effects of bleaching on dental substrates, thereby improving the longevity and efficacy of adhesive restorations. Incorporating antioxidants after bleaching can reverse adhesion failures caused by hydrogen peroxide’s mechanism of action, effectively restoring the bond strength of restorative materials [36,47].

Importantly, the clinical implications of these findings highlight the potential of quercetin as an adjunct in dental bleaching protocols, especially for patients requiring immediate adhesive procedures. Compared to other antioxidants commonly used in dentistry, such as sodium ascorbate, quercetin exhibits not only comparable efficacy but also additional biological advantages due to its multifunctional properties [22,23]. At the molecular level, quercetin modulates oxidative stress by upregulating antioxidant enzymes (e.g., SOD, CAT, GPx), inhibiting the NF-κB signaling pathway, and enhancing mitochondrial function, thereby contributing to cellular protection and tissue homeostasis [48]. Future research should focus on evaluating whether quercetin-containing bleaching gels improve the adhesion of restorative materials to dental substrates compared to conventional bleaching agents. Additionally, studies should confirm their potential to prevent bleaching-induced alterations in dental structures, ultimately promoting the integrity and longevity of restorations following bleaching procedures.

This study has some limitations worth noting. It employed an in vitro design utilizing artificially pigmented bovine teeth. Although this protocol is widely accepted in tooth whitening research and provides results comparable to clinical observations [49], and although bovine teeth are commonly used as substitutes in dental research due to their availability and ease of standardization, it is important to acknowledge that there are differences in physicochemical properties between bovine and human teeth, including variations in enamel and dentin composition, microstructure, porosity, permeability, and hardness. Moreover, the study focused on experimental whitening formulations aimed at enhancing procedural safety. However, laboratory results may not fully translate to clinical settings, where factors such as the acquired pellicle, varied pigmentation types, use of fluoridated products, and potential reductions in tooth sensitivity could affect outcomes. Given that the study was conducted under in vitro conditions, the potential influence of in vivo factors, such as dietary habits (e.g., the consumption of tea, wine, or other staining substances), should also be considered. These factors could alter the bleaching efficacy and enamel response in real-world scenarios. Furthermore, it is important to note that no direct cytotoxicity data were obtained in this study. This represents another limitation, as cytocompatibility is crucial in evaluating the biological safety of new therapeutic agents. Although quercetin appears to reduce peroxide diffusion, its potential protective effect on pulp tissues has yet to be confirmed. In addition, the physicochemical properties of the quercetin-containing gels, such as viscosity, stability, and rheological behavior, were not evaluated in the present study. Therefore, future studies should include cytotoxicity assays, in vivo evaluations, and the physical characterization of the bleaching gels to fully establish the clinical relevance and safety of quercetin-containing bleaching protocols. Despite these limitations, this study breaks new ground by identifying a gap in the literature regarding quercetin, especially in the context of tooth whitening. The results suggest that incorporating quercetin into bleaching gels holds promise. It is therefore important to evaluate the efficacy of these gels in clinical trials, as this could aid in advancing whitening techniques with improved biocompatibility and esthetic outcomes, minimizing adverse effects and promoting safer dental bleaching practices.

## 3. Conclusions

Within the limitations of this in vitro study, the incorporation of 1% quercetin into a 35% hydrogen peroxide bleaching gel effectively prevents enamel surface microhardness and roughness, while also reducing the transamelodentinal diffusion of H_2_O_2_. Importantly, the addition of quercetin did not compromise the whitening efficacy. These findings suggest that quercetin may enhance the safety profile of bleaching gels by minimizing enamel alterations, thereby offering a more biocompatible approach to dental whitening. Nonetheless, further studies such as in vivo and long-term clinical trials are essential to confirm these findings and evaluate their broader clinical applicability.

## 4. Materials and Methods

### 4.1. Experimental Design

For the study, enamel/dentin discs were obtained from bovine incisor teeth. To inhibit bacterial growth, the cleaned teeth were immersed in a 0.1% thymol solution and stored at approximately 4 °C until the experimental phase commenced. The sample size of 12 enamel/dentin discs per group was established based on a prior study [30], with surface and longitudinal hardness as the primary endpoints. The calculations considered a mean difference between the groups, an α error of 5%, and a β error of 10%. As a result, 60 discs were assigned for color change assessment, an equal number for hardness and roughness assessments, and another set for evaluating H_2_O_2_ transamelodentinal diffusion. The discs were randomly distributed into five experimental groups (*n* = 12), according to the treatments: negative control—no treatment (NC); 35% hydrogen peroxide (HP); HP + 0.25% quercetin (HP/0.25%QC); HP + 0.5% quercetin (HP/0.5%QC); and HP + 1% quercetin (HP/1%QC). The treatment was carried out in three sessions of 40 min each, at seven-day intervals. Between sessions, the specimens were kept in artificial saliva, which was renewed daily. After the treatments, the analyses were assessed.

### 4.2. Preparation of Enamel/Dentin Discs

**Figure 4:**
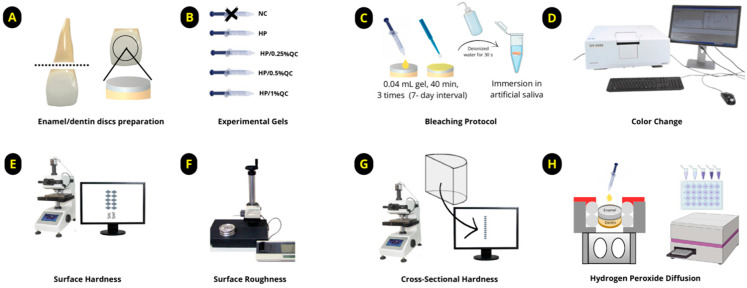
Diagram illustrating the experimental design of the study. (**A**) Preparation of enamel/dentin discs. (**B**) Formulation of experimental gels. (**C**) Bleaching protocol. (**D**) Analysis of color change. (**E**) Measurement of surface hardness. (**F**) Measurement of surface roughness. (**G**) Cross-sectional hardness (KHN × μm). (**H**) Transamelodentinal diffusion of hydrogen peroxide analysis.

The samples were prepared using bovine teeth collected from animals aged between two and three years. After extraction, the incisor teeth were thoroughly cleaned and preserved in a thymol solution [10,31]. The roots were sectioned, and discs were obtained from the vestibular surface using a diamond-tipped glass cutting tool, ensuring continuous irrigation throughout the process (Figure 4A). The discs were polished to a final thickness of 3.5 mm (1.3 mm of enamel and 2.2 mm of dentin) using a grinder–polisher (BETA Polisher, Buehler, Lake Bluff, IL, USA) equipped with 400- and 600-grit silicon carbide sandpaper (Extec Corp., Enfield, CT, USA), followed by cleaning in an ultrasonic bath (Unique USC 1400, Indaiatuba, SP, Brazil) for 20 min [10].

### 4.3. Formulation and Determination of Experimental Groups

The gels were formulated as colorless (unpigmented) and stored in syringes for application. Each formulation was prepared step by step as follows: initially, a 12% (*w*/*v*) Carbopol solution (Carbopol 960; Pharmacy Apothicario, Araçatuba, SP, Brazil) was prepared as the thickening agent. Then, H_2_O_2_ (Pharmacy Apothicario) at a final concentration of 35% (*v*/*v*) was added as the bleaching agent [30]. Quercetin (Sigma, St. Louis, MA, USA) was incorporated into the formulations at concentrations of 0.25%, 0.5%, or 1% (*w*/*v*), depending on the experimental group. Deionized water was added in a sufficient quantity (q.s.) to complete the formulation. Finally, sodium hydroxide (NaOH, Sigma) was used to adjust the pH of the gels to approximately 7.0 (Table 2). The five experimental groups were defined as follows: (1) negative control—without treatment (NC); (2) 35% hydrogen peroxide (HP); (3) HP+ 0.25% quercetin (HP/0.25%QC); (4) HP + 0.5% quercetin (HP/0.5%QC); (5) HP + 1% quercetin (HP/1%QC) (Figure 4B).

### 4.4. Color Change Analysis

#### 4.4.1. Preliminary Selection and Allocation of Enamel/Dentin Discs

After the discs were prepared, a first color measurement (initial) was taken following the Commission Internationale de l’Eclairage (CIE) standards, which enable accurate color definition in a three-dimensional space. A spectrophotometer (Model UV-2450, Shimadzu, Kyoto, Japan) was used, applying the CIELab color system [8,10]. The L* a* b* values were recorded, and an average was calculated for all the samples. From this, the 120 dental discs with L* a* b* values most closely matching the average, within a 5% variation, were selected for further examination.

#### 4.4.2. Staining Process of Enamel/Dentin Discs

Sixty samples (*n* = 12 per group) were stained using a black tea infusion (1 mL—Chá Matte Leão, Curitiba, PR, Brazil), with the samples placed in microtubes (Eppendorf, Stevenage, UK) at room temperature following established protocols [10,49]. The pigmentation process lasted six days, with the tea infusion refreshed daily. After pigmentation, a secondary selection of the discs was conducted using the same procedure as initially described.

#### 4.4.3. Bleaching Procedure

After selecting the 60 enamel/dentin discs, the samples were randomized and subjected to the bleaching protocol. Each disc received 0.04 mL of gel, which was allowed to remain in contact for 40 min per session. The bleaching procedure was performed weekly, with a 7-day interval between sessions, totaling three treatment sessions. After each application, the gels were removed using gauze, followed by a 30 s rinse with deionized water to eliminate any residual product (Figure 4C). During the experimental period, the samples were stored individually in artificial saliva (pH 7.0) [10,24], which was replaced daily until the protocol was completed.

#### 4.4.4. Color Measurements

Enamel/dentin discs were inserted in black silicone holders to ensure uniform light exposure during spectrophotometric analysis using the UV-2450 (Shimadzu, Kyoto, Japan). The equipment operated within a wavelength range of 400–700 nm, utilizing D65 illumination and a 45/0° geometry. Shade measurements were performed at distinct time points: baseline (before staining), post-staining, 24 h after each bleaching session, and 14 days following the completion of bleaching (Figure 4D). The evaluation of color alterations involved calculating the differences in lightness (ΔL*), red–green (Δa*), and yellow–blue (Δb*) coordinates. The overall color change was quantified using the ΔE_ab_ formula: ΔE_ab_ = √[(ΔL*)^2^ + (Δa*)^2^ + (Δb*)^2^] [10,49]. Additionally, the Whitening Index for Dentistry (ΔWI_D_) was determined using the following equation [50]: ΔWI_D_ = 0.511L* − 2.324a* − 1.100b*. For a more nuanced analysis, the CIEDE2000 color difference formula (ΔE_00_) was applied [51], ΔE_00_ = √[(ΔL/SL)^2^ + (ΔC/SC)^2^ + (ΔH/SH)^2^ + RT × (ΔC/SC) × (ΔH/SH)], where SL, SC, and SH are weighting functions for lightness, chroma, and hue differences, respectively, and RT is a rotation term accounting for interactions between chroma and hue differences. In this context, perceptibility and acceptability thresholds were set at 0.81 and 1.81, respectively [10,52].

### 4.5. Assessment of Surface Roughness, Surface Hardness, and Cross-Sections

The enamel surfaces of 60 enamel/dentin discs were prepared by leveling and polishing according to established protocols [10,30]. After polishing, the initial surface hardness was determined with a Micromet 5114 hardness testing device (Buehler, Lake Bluff, IL, USA), utilizing a Knoop diamond indenter under a 25 g load applied for 10 s [10]. Surface roughness was assessed with a profilometer (SJ-401, Mitutoyo, Kawasaki, Japan), which was equipped with a 2 mm radius stylus. The device operated at a fixed speed of 0.1 mm/s, with a 5 N load applied and a 0.25 mm cut-off value [10]. Discs with initial SH values ranging from 355.0 to 376.8 for Knoop hardness number (KHN) were randomly assigned into 5 experimental groups, with 12 discs per group.

After completing the bleaching procedure, the final surface hardness (Figure 4E) and roughness (Figure 4F) were re-evaluated. The discs were then bisected, with one half embedded in acrylic resin and polished [30] for cross-sectional hardness analysis (Figure 4G). Knoop hardness was measured at multiple depths (5–180 μm) from the surface, using a load of 5 g for 10 s. The integrated hardness (KHN × μm) was determined through the application of the trapezoidal method [8,10]. To obtain the integrated loss of subsurface hardness (ΔKHN; KHN × μm), the integrated hardness for sound enamel was subtracted from the integrated area of the subsurface regions in the enamel [8,10,31].

### 4.6. Transamelodentinal Diffusion of H_2_O_2_ Assay

The discs (*n* = 12 per group) were placed into artificial pulp chambers and arranged in 24-well culture plates. The dentin surfaces were maintained in direct contact with a sodium acetate buffer solution (1 mL per chamber) to simulate experimental conditions [10]. The bleaching was performed in a single application, following the procedure described earlier, and the chambers were kept in a humidified environment at 37 °C. To quantify H_2_O_2_ diffusion, the colorimetric method described by Mottola et al. [53] was employed. The discs were exposed to bleaching gels for 40 min, and diffusion was quantified using a standard curve ranging from 0.5 to 5.0 μg/mL for H_2_O_2_ concentrations. The solutions were prepared with 1 mL of leucocrystal violet (Sigma, St. Louis, MA, USA), 50 μL of peroxidase enzyme (Sigma, St. Louis, MA, USA), and 3 mL of deionized water. H_2_O_2_ levels in the samples were measured via spectrophotometry at 596 nm (Figure 4H).

### 4.7. Statistical Analysis

Statistical analysis was performed using Sigmaplot^®^ version 12.0 for Windows (Systat Software Inc., San Jose, CA, USA), with the significance level set at 5%. Analyses were performed for color (ΔE_ab_, ΔWI_D_, ΔE_00_), surface hardness (SH), and surface roughness (Ra), considering both the treatment and time as factors. For %SH, cross-sectional hardness (ΔKHN), and H_2_O_2_ diffusion, the values were treated as dependent variables, with treatments as the factor. The data were assessed for normality using the Shapiro–Wilk test and for homogeneity of variances using Levene’s test. Upon confirming that the assumptions were met, ANOVA was conducted, followed by the Student–Newman–Keuls post hoc test for pairwise comparisons.

## Figures and Tables

**Table 1:** Mean values (SD) for roughness (Ra), surface hardness (SH), and percentage of surface hardness change (%SH), according to treatments (*n* = 12).

Treatments	Variables				
	Ra		SH		
	Initial	Final	Initial	Final	%SH
NC	0.068 aA (0.015)	0.070 aA (0.018)	368.1 aA (5.7)	369.7 aA (3.5)	0.4 a (1.0)
HP	0.071 aA (0.018)	0.116 bB (0.012)	368.1 aA (6.4)	307.4 bB (3.1)	−16.5 b (1.4)
HP/0.25%QC	0.068 aA (0.020)	0.088 cB (0.017)	368.3 aA (6.2)	347.6 cB (3.4)	−5.6 c (1.7)
HP/0.5%QC	0.071 aA (0.024)	0.084 cB (0.023)	368.0 aA (6.0)	354.5 cB (4.1)	−3.6 c (1.7)
HP/1%QC	0.069 aA (0.014)	0.073 aA (0.011)	368.0 aA (6.1)	365.5 aA (4.7)	−0.7 a (0.6)

Distinct superscript lowercase letters indicate statistical differences among bleaching gels in each variable (Student–Newman–Keuls test, *p* < 0.001). Different capital letters indicate the difference between the analysis moment (initial and final) for Ra and SH variables (Student–Newman–Keuls; *p* < 0.05).

**Table 2:** Experimental dental bleaching gels, composition, and pH.

Experimental Gels	Composition	**pH**
HP	35% HP, carbopol^®^ polymers thickeners, and NaOH 4.0 Mol/L to adjust the pH	7.02 (0.2)
HP/0.25%QC	35% HP, carbopol^®^ polymers thickeners, 0.25% quercetin, and NaOH 4.0 Mol/L to adjust the pH	7.03 (0.1)
HP/0.5%QC	35% HP, carbopol^®^ polymers thickeners, 0.5% quercetin, and NaOH 4.0 Mol/L to adjust the pH	7.05 (0.3)
HP/1%QC	35% HP, carbopol^®^ polymers thickeners, 1% quercetin, and NaOH 4.0 Mol/L to adjust the pH	7.03 (0.2)

Abbreviations: HP, hydrogen peroxide. QC, quercetin. NaOH, sodium hydroxide.

## Data Availability

The original contributions presented in this study are included in the article. Further inquiries can be directed at the corresponding author.
